# A case report of greater saphenous vein thrombosis in a patient with coronavirus (COVID-19) infection

**DOI:** 10.1186/s40794-021-00131-9

**Published:** 2021-03-03

**Authors:** Negin Hesam-Shariati, Poya Fatehi, Fardin Fathi, Morteza Abouzaripour, Mohammad Bakhtiar Hesam Shariati

**Affiliations:** 1 School of Medical Sciences, Faculty of Medicine, University of New South Wales, Sydney, Australia; 2 Department of Radiology, Tohid Hospital, Kurdistan University of Medical Sciences, Sanandaj, Iran; 3 Cellular and Molecular Research Center, Research Institute for Health Development, Kurdistan University of Medical Sciences, Sanandaj, Iran; 4 Department of Anatomical Sciences, Faculty of Medicine, Kurdistan University of Medical Sciences, Sanandaj, Iran

**Keywords:** Superficial vein thrombosis, Coronavirus, COVID-19, Ultrasonography, Case report

## Abstract

In December 2019, the World Health Organization (WHO) announced a series of pneumonia cases caused by an unknown origin, discovered in Wuhan, China. A dangerous virus called severe acute respiratory syndrome coronavirus 2 (SARS-CoV-2) caused a disease named acute respiratory syndrome, which was later popularly called coronavirus infection (COVID-19). Patients with acute COVID-19 are at high risk of thrombosis in various blood vessels due to hypercoagulability, blood stasis, and endothelial damage. In this study, we present a case report of a patient with COVID-19, who was hospitalized in one of the hospitals in Sanandaj, Iran. There were symptoms of fever, chills, muscle aches, cough, and tachycardia. Laboratory tests showed high levels of CRP, ESR, Ferritin CLIA, LDH and D-Dimer in this patient. Doppler ultrasound of the patient also revealed an abnormal finding, thrombosis in the right greater saphenous vein. This suggests that COVID-19 may lead to other negative effects through damage to blood vessels.

## Introduction

Coronavirus has been widespread around the world since early 2020. The disease is highly contagious and, in severe cases, can lead to acute respiratory syndrome or organ failure [1, 2]. In January 2020, the World Health Organization (WHO) announced the outbreak of the disease as a “public health emergency of international concern” [3]. To date, the virus has led to an unprecedented global health crisis that has resulted in over 2 million death worldwide [4].

Several studies have shown that superficial vein thrombosis (SVT) is a common venous disease that appears to be medically benign but can cause serious complications and may be associated with complications such as deep vein thrombosis (DVT) and pulmonary embolism (PE) [5, 6]. The prevalence of SVT is estimated to be about 3 to 11%, while the incidence of thrombosis in the greater saphenous vein (GSV) is about 60 to 80% of the time [7, 8]. The proximity of the greater saphenous vein to the saphenofemoral junction (SFJ) increases the possibility of displacement of blood clots and their entry into the deep venous system and as a result makes SVT a serious concern [9].

There is a risk for venous and arterial thrombosis in patients with SARS-Cov2 due to excessive coagulation status, blood stasis, and damage to vascular endothelial cells in this condition [10]. As the clinical signs of venous and arterial thrombosis are ambiguous, it is very important to use imaging techniques such as Doppler ultrasound and computed tomography (CT) angiography to prevent catastrophic complications such as pulmonary embolism and mortality [11]. In this case report, the course of a patient with coronavirus is described.

## Case presentation

**Fig. 1:**
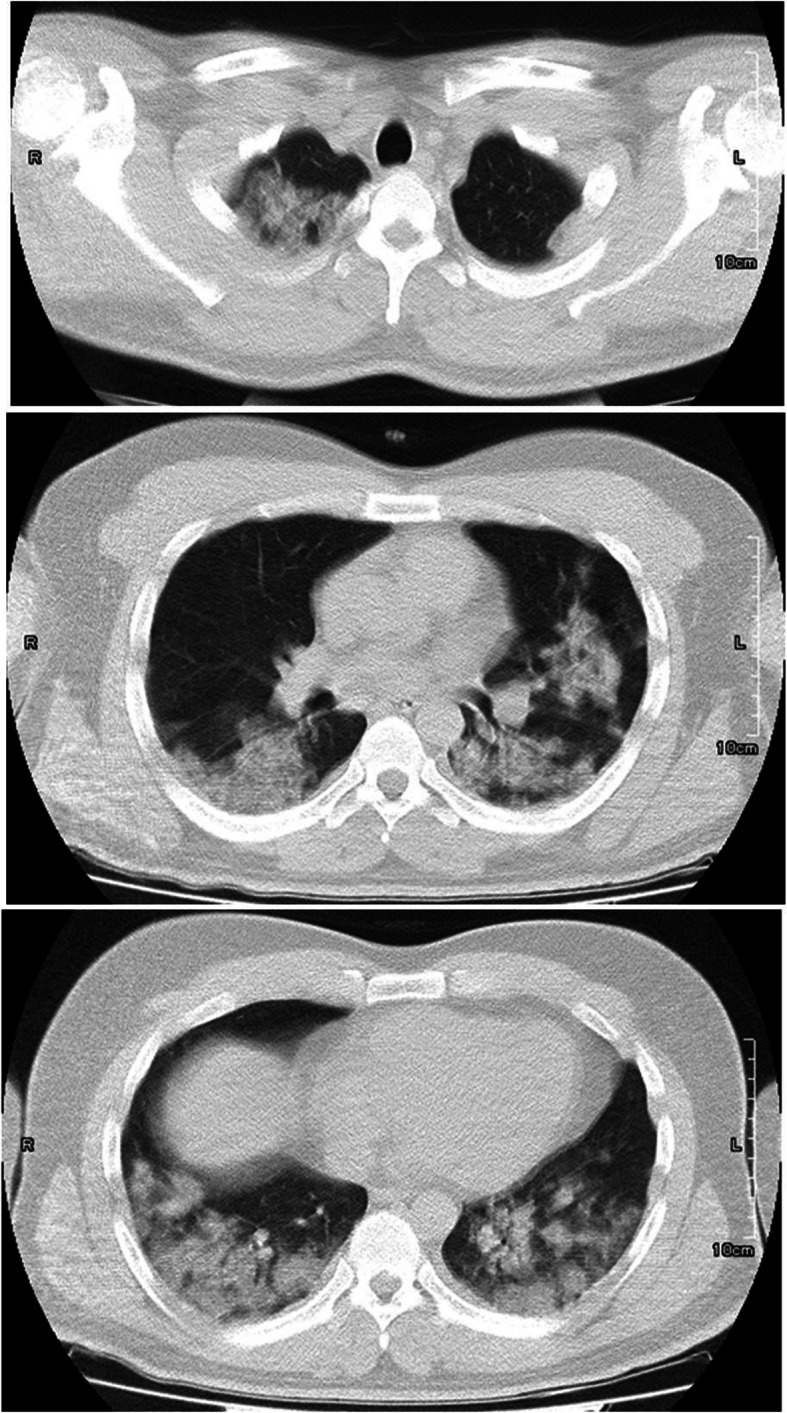
Axial without contrast-enhanced chest computed tomography (CT) image showing a coronavirus disease (COVID-19) infection

A 40-year-old man with one-week symptoms of cough, fever, fatigue, muscle aches, diarrhea, palpitations, and shortness of breath but no chest pain was admitted to Tohid Hospital, Sanandaj, Iran. Before admission, the patient was diagnosed with COVID-19 by an infectious disease specialist based on the initial symptoms. Both CT scans (Fig. 1) and the real-time reverse transcriptase-polymerase chain reaction (RT-PCR) confirmed the infection. The patient had no history of underlying diseases such as diabetes, heart disease, hypertension, or cancer. At the hospital’s emergency department, the physical examinations showed that the patient had an irregular heart rate of 145 beats/min, blood pressure of 82/75 mmHg, temperature of 38.4 °C, respiratory rate of 26 breaths/min, and oxygen saturation of 89%. Paraclinical and laboratory results showed that routine blood tests, renal function, and electrolytes were completely normal. The influenza A and B antigen tests were also negative. However, the other laboratory findings were all abnormal, which are briefly listed in Table 1. In CT scans of the lungs (Fig. 1), bronchovascular marking is evident. Additionally, multiple foci of parenchymal turbidity and ground-glass opacity were observed with greater density at the margins and at the base of the lungs. Therefore, the patient was started on medical treatment with Naproxen, Hydroxychloroquine, Famotidine, Zinc, Neurobion, and anticoagulants by injecting heparin and taking acetylsalicylic acid tablets.

**Table 1:** The results of laboratory findings

	Test Name	Unit	Reference range	Results	Flag
**1**	BUN	mg/dl	6–20	32	Hi
**2**	CPK	IU/L	male: 0–171	55	
**3**	LDH	U/L	235–470	510	Hi
**4**	Na(ser)	mEq/L	138–145	134	LOW
**5**	K(ser)	mEq/L	3.6–5.9	3.8	
**6**	Cr	mg/dl	male:0.8–1.3 mg/dl	0.7	LOW
**7**	CRP	mg/l	0–6	30	Hi
**8**	ESR	mm	5–12	18	Hi
**9**	Ferritin CLIA	ng/mL	50–434	511	Hi
**10**	**D-Dimer(CLIA-Siemens)**	**ng/mL**	**< 885**	**> 7500**	**Hi**

**Fig. 2:**
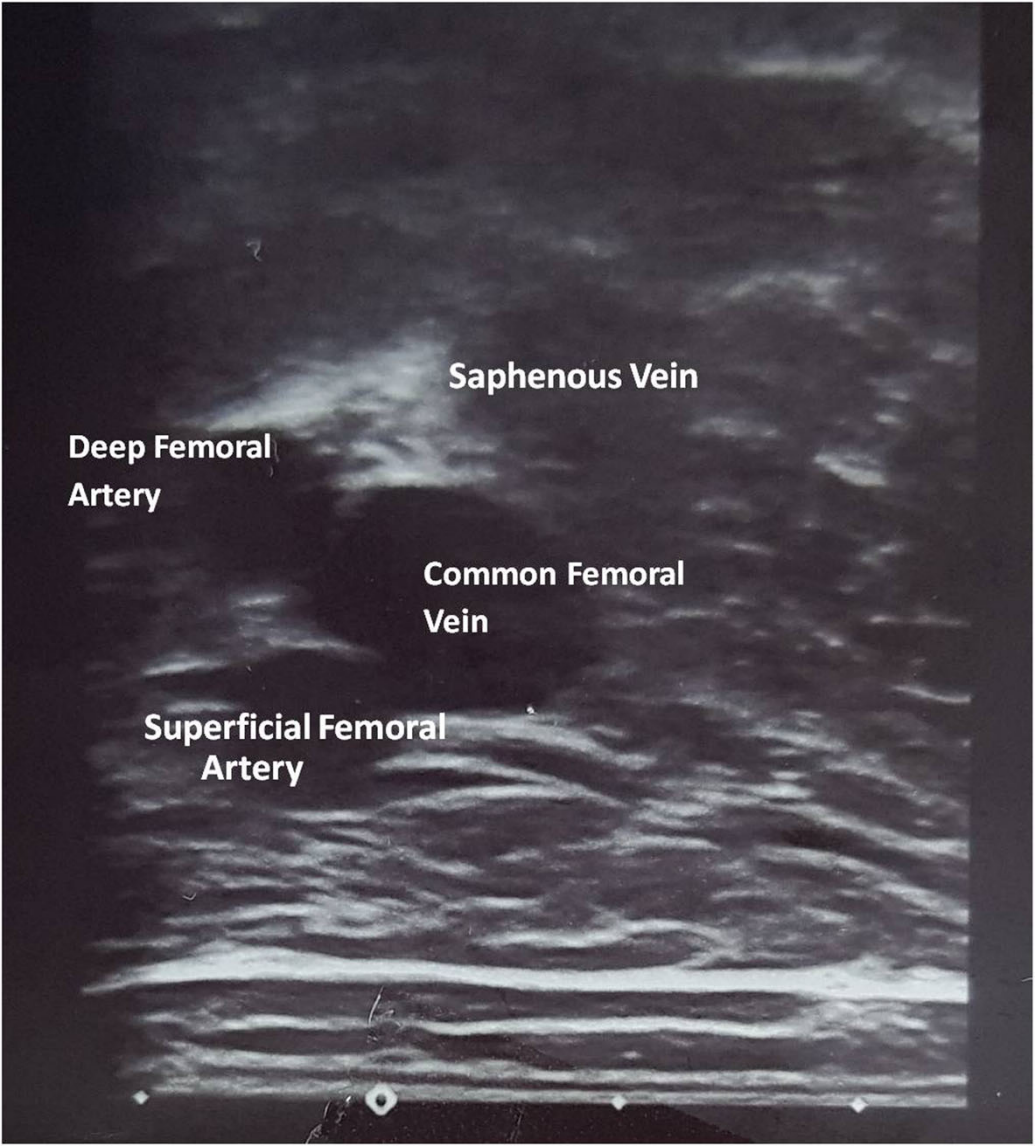
Doppler ultrasound images of the lower right limb showing a superficial vein thrombosis (SVT). Anterior accessory saphenous vein (AASV), great saphenous vein (GSV), common femoral vein (CFV), and common femoral artery (CFA) in the Doppler ultrasound images of the lower right limb

Three days after hospitalization, Doppler ultrasound was performed on the lower limb due to numbness and tingling (paresthesia) in the right leg, in addition to swelling, redness, pain, and sensitivity to touch. Examination of the main veins of both lower limbs showed no evidence of occlusion in the external iliac, common femoral, popliteal, anterior and posterior tibialis, and peroneal. However, more detailed examination revealed that thrombosis was evident at the beginning of the greater saphenous vein of the right leg from distal to proximal (Fig. 2). The patient was discharged after 12 days of hospitalization with complete recovery from COVID-19. The anticoagulation treatment for the GSV thrombosis was continued for the patient, and no negative side effect caused by SVT was reported after the treatment.

## Discussion

In this case report, we presented a patient with COVID-19 who was hospitalized in Tohid Hospital, Sanandaj, Iran and later was diagnosed with a thrombosis in his right GSV. This patient had common COVID-19 symptoms such as fever, dry cough, shortness of breath, and muscle pain [12] but no risk factor for SVT. Paraclinical tests and CT scans of the chest confirmed the COVID-19 diagnosis, and although there was no obvious evidence of SVT, detailed examination by Doppler ultrasound revealed a thrombosis in the patient’s GSV.

Studies have shown that the most important and stable hemostatic disorders associated with COVID-19 include mild thrombocytopenia [13] and an increase in D-dimer amount [14]. There is evidence showing thrombotic abnormalities, in addition to abnormalities in the function of various organs in patients with COVID-19 [15], which lead to higher mortality. However, as far as we can ascertain there are few reports of SVT and its side effects in patients with COVID-19.

Pathophysiologically, patients with COVID-19 may have a higher risk for developing venous thrombosis, usually due to diarrhea, hypotension, recurrent long-term infections, and dehydration [16]. Therefore, in patients with coronavirus, assessing the risk of DVT and SVT are essential to reduce complications and mortality risk. Studies have shown that patients prone to DVT usually have one of the following criteria: age over 75, respiratory and heart failure, history of previous thrombosis, acute onset of chronic pulmonary obstruction, acute cerebral infarction, malignant tumor, limb varicose veins, obesity, chronic kidney disease, inflammatory bowel disease, and more than 3 days of bed rest [17].

In one study on a patient with COVID-19, CT images of angiography showed signs of acute cerebral infarction and DVT in both lower limbs [18]. In our case report, the patient was suspected of having thromboembolism due to having similar lesions on his leg, however after a CT scan of his chest, his diagnosis with COVID-19 was confirmed, while there was no evidence of pulmonary thromboembolism. The physicians at the hospital also suspected SVT and DVT due to numbness, swelling, and pain in the right leg, which were examined by Doppler ultrasound of all blood vessels, including the common iliac, small saphenous, and greater saphenous. Since many studies have reported respiratory distress along with other clinical evidence of venous thrombosis, pulmonary embolism should be suspected [19, 20].

A recent study on the intensive care unit (ICU) patients with COVID-19 found that the rate of thrombotic disorders in these patients is 31% [21]. Further, medical images have shown that 27% of such thrombotic disorders are due to venous thromboembolism, 3.7% to arterial thrombosis, and 81% to pulmonary embolism, which is the most common complication of thrombosis in ICU patients [21]. The possible reasons for venous thrombosis may include the fact that COVID-19 attacks the human body via the 2-angiotensin converting enzyme, which is found in various blood vessels and organs of the body [22]. Ultimately, coronaviruses cause cytokine waterfalls, including IL2, IL7, IL10, GCSF, IP10, MCP1, MIP1A, and TNFα in the body, which can increase the risk of complications such as blood clots. This cytokine storm can be associated with the severity of the disease and its negative consequences [23, 24]. Blood clots formed in DVT may also have a variety of causes, including vascular damage, surgery, special medications, and limited mobility [25], but the exact cause of COVID-19-induced DVT is still unknown [26].

## Conclusion

COVID-19 is an emerging source of venous thrombosis due to factors such as excessive coagulation, blood stasis, and endothelial damage. The main mechanism of SVT and DVT formation due to COVID-19 is unknown and has not yet been examined. Although COVID-19 cases presented with SVT and DVT are rare, recognizing SVT and DVT as potential complications of COVID 19 infection will be of great value. Imaging techniques such as CT, MRI, and ultrasound can confirm the diagnosis of SVT and DVT. Due to the possibility of COVID-19 infection in patients presenting with venous thrombosis or other thromboembolic diseases, it seems important to consider the presence of COVID-19 in their diagnosis.

## Data Availability

I have presented the data of the patient in the manuscript as a Table. I have submitted the figures separately as figures.
